# 2,3-Dehydrokievitone combats methicillin-resistant *Staphylococcus aureus* infection by reducing alpha-hemolysin expression

**DOI:** 10.3389/fmicb.2022.969215

**Published:** 2022-08-23

**Authors:** Hangqian Yu, Jingyu Liu, Li Wang, Shuhan Guan, Yajing Jin, Jianze Zheng, Hua Xiang, Dacheng Wang, Dianfeng Liu

**Affiliations:** ^1^College of Animal Science, Jilin University, Changchun, China; ^2^Changchun University of Chinese Medicine, Changchun, China; ^3^College of Animal Medicine, Jilin Agricultural University, Changchun, China

**Keywords:** antivirulence, α-hemolysin, methicillin-resistant *Staphylococcus aureus*, pneumonia, 2,3-dehydrokievitone

## Abstract

Due to powerful drug resistance and fatal toxicity of methicillin-resistant *Staphylococcus aureus* (MRSA), therapeutic strategies against virulence factors present obvious advantages since no evolutionary pressure will induce bacterial resistance. Alpha-hemolysin (Hla) is an extracellular toxin secreted by *Staphylococcus aureus* and contributes to bacterial pathogenicity. Herein, we identified a natural product 2,3-dehydrokievitone (2,3-DHKV) for inhibiting Hla activity of MRSA strain USA300 but not affecting bacteria growth. 2,3-DHKV significantly decreased hemolysin expression in a dose-dependent manner, but it did not potently neutralize hemolysin activity. Subsequently, cellular thermal shift and heptamer formation assays confirmed that 2,3-DHK affects hemolytic activity through indirect binding to Hla. RT-qPCR and western blot revealed that 2,3-DHKV suppressed Hla expression at the mRNA and protein levels, and further decreased accessory gene regulator A (*agrA*) transcription levels. We also observed that 2,3-DHK significantly attenuated the damage of A549 cells by *S. aureus* and reduced the release of lactate dehydrogenase (LDH). Moreover, in the MRSA-induced pneumonia mouse model, 2,3-DHK treatment prolonged the life span of mice and reduced the bacterial load in the lungs, which significantly alleviated the damage to the lungs. In summary, this study proved that 2,3-DHK as a Hla inhibitor is a potential antivirulence agent against MRSA infection.

## Introduction

*Staphylococcus aureus* (*S. aureus*) is a pathogenic bacterium with a wide range of pathogenic sites, which causes a variety of invasive and toxin-related diseases (Hodille et al., 2017). With the evolution of multidrug-resistant pathogens, especially the widespread methicillin-resistant *Staphylococcus aureus* (MRSA) strains, the post-antibiotic era is burgeoning, and bacterial infection has once again become a major challenge to human health and safety worldwide (George et al., 2008). MRSA has been ranked as one of nine bacterial species of international concern by the World Health Organization (WHO). MRSA not only accounts for 20%–80% of all *S. aureus* infections, but also has the highest case fatality rate of any antibiotic-resistant pathogen (Köck et al., 2010; Wu et al., 2019). At present, antibiotic therapy for MRSA is limited due to the strong antibiotic resistance. Combined with poor clinical effects, the minimal inhibitory concentration (MIC) keeps constantly increasing, non-negligible side effects, and expensive pharmacy expenses (Rodvold and McConeghy, 2014). As a result, it is critical to pursue novel therapeutic strategies that can effectively compensate for the shortcomings of traditional antibiotics.

Alternative strategies to tackle infections of *S. aureus* have been reported include phage therapy, antivirulence therapy, antimicrobial peptides, vaccines, and others (Iskandar et al., 2022). *S. aureus* utilizes a variety of virulence factors to invade the host and allow it to survive, including binding to host cells and facilitating colonization to kill or change signal transduction in host cells. Interference with the expression of bacterial virulence factors may allow enough time for the host immune system to restrain bacteria internalize and alleviate the symptoms of the host.

Alpha-hemolysin (Hla) is a 319 amino acid-sized beta-barrel pore-forming toxin that is regulated by the accessory gene regulator (Agr) system and can form transmembrane oligomeric β-barrel heptameric channels leading to host cell death (Surewaard et al., 2018). For example, Hla could severely destroy host erythrocytes and platelets and pose a variety of pathological injuries (von Hoven et al., 2019). Multiple studies have shown that reducing Hla expression could significantly alleviates the injury and mortality of MRSA infected mice (Wu et al., 2019; Yin et al., 2022). In addition, Hla is a major virulence factor upon which *S. aureus* depends for survival in the host immune system. In previous studies, Hla enhances the internalization and viability of *S. aureus* in mast cells by regulating expression of β1integrin. In addition, Hla also helps *S. aureus* evade killing by macrophages through the dependence of NLRP3 on mitochondrial trafficking (Cohen et al., 2018). Therefore, Hla is a potential target to inhibit the virulence of *S. aureus*.

The natural compound has emerged as a powerful resource reservoir for inhibitors of virulence factors because of its wide range of biological activities and rich source. In this study, we identified that 2,3-dehydrokievitone (2,3-DHKV) was able to potently inhibit hemolysin activity with no killing effect against *S. aureus*. 2,3-DHKV, an isoflavonoid extracted from the roots of yellow lupin, has cytotoxic activity against prostate, cervical, and ovarian adenocarcinoma cells and can effectively induce cancer cell apoptosis (Tsukayama et al., 1994; Yoon et al., 2016).

Herein, we performed bacterial phenotyping assays on USA300 to observe the effects of 2,3-DHKV on Hla expression and function. More importantly, the mouse model of MRSA-induced lethal pneumonia was established to investigate whether Hla has protective and therapeutic effects on MRSA-induced injury. This study will provide an experimental basis and rationale for the application of the natural product 2,3-DHKV in the field of anti-MRSA infection.

## Materials and methods

### Bacterial strains, reagents, and growth conditions

MRSA USA300 (BAA-1717), methicillin-sensitive *S. aureus* (MSSA) Newman, and human lung adenocarcinoma cells A549 were all brought from American Type Culture Collection (ATCC, Manassas, United States). Clinical strain of SA1B3G was isolated from the Affiliated Hospital of Liaoning University of Chinese Medicine (Shenyang, Liaoning) and kept in our laboratory. Before the experiment, 16*sRNA* identification was first performed, and then PCR was used to distinguish MRSA and MSSA by identifying the *mecA* gene. Furthermore, the MIC of cefoxitin against clinical isolates was tested again. The drug sensitivity results were judged according to the criteria established by the American Society for Clinical Laboratory Standardization. MRSA was determined by a minimum inhibitory concentration (MIC) of cefoxitin MIC ≥ 8 μg/ml (Clinical and Laboratory Standards Institute, 2014). Ultimately, SA1B3G was identified as a MRSA strain. The clinical isolate was stored at −80°C in 20% glycerol-rich BHI and passaged twice on trypticase soy agar prior to the experiment. All bacteria were grown in Tryptic Soy Broth (TSB) or Luria-Bertani (LB) broth and placed in a 37°C environment on a shaker at 220 rpm/min unless otherwise stated. A549 cells were grown in 1,640 medium with 10% fetal bovine serum. The natural product 2,3-DHKV was purchased from BioBioPha (Yunnan, China) at 98% purity and dissolved with dimethyl sulfoxide (DMSO, Sigma, United States). Other reagents were supplied by Sangon Biotech (Shanghai, China).

### Construction, expression, and purification of the Hla protein

A prokaryotic expression vector for pET28a-hla was constructed to obtain the recombinant Hla protein. Briefly, the *hla* gene fragment was amplified from *S. aureus* USA300 genomic, and the primers used are in Table 1. The PCR product was then digested with *BamH*I and *Xho*I and ligated to pET28a after ligation with T4 DNA ligase (Takara, Dalian, China) with the same double digestion. The recombinant plasmids were then transformed into *E. coli* DH5α. After being identified through sequencing, it was transformed into *E. coli* BL21 (DE3) to induce Hla protein expression. When *E. coli* was cultured to an OD_600_ of 0.8, isopropyl-beta-D-thiogalactopyranoside (IPTG, 0.5 mM) was added to induce the expression of Hla. Bacteria were then collected and lysed by sonication, the supernatant was collected and the HIS-Select nickel affinity gel (Beyotime, Shanghai, China) system was used to purify Hla protein.

**Table 1:** Primers used in this study.

Primer name	Sequences (5′–3′)
*Hla*-F	CGCGGA TCCGCAGATTCTGATATTAATATTAAAAC
*Hla*-R	CCGCTCGA GTTAATTTGTCATTTCTTCTTTTTC
rt-*16s*RNA-F	TACACACCGCCCGTCACA
rt-*16s*RNA-R	CTTCGACGGCTAGCTCCAAAT
rt-*hla*-F	ACAATTTTAGAGAGCCCAACTGAT
rt-*hla*-R	TCCCCAATTTTGATTCACCAT
rt-*agrA*-F	GCAGTAATTCAGTGTATGTTCA
rt-*agrA*-R	TATGGCGATTGACGACAA
rt-*PVL*-F	GAGGTGGCCTTTCCAATACAAT
rt-*PVL*-R	CCTCCTGTTGATGGACCACTATTA
rt-*psmα*-F	TATCAAAAGCTTAATCGAACAATTC
rt-*psmα*-R	CCCCTTCAAATAAGATGTTCATATC
rt-*sspA*-F	CTACAACTACACCGGAAGCAATAAA
rt-*sspA*-R	ACAGACAAACAGCAAACACCTAAGA

### MIC assay

A microdilution susceptibility assay was used to examine the MIC of compound 2,3-DHKV against *S. aureus* USA300. Following the procedures outlined in the Clinical Laboratory Standards Institute’s 2017 guideline (El-Shaer et al., 2017), the USA300 has first diluted 1,000-fold with Caton-adjusted Mueller-Hinton Broth (CAMHB) medium and cultured to an OD_600_ of 0.8 (Wexler, 1991). Each well added 100 μl of USA300 containing 1 × 10^5^ CFUs/ml. The 2,3-DHKV was then added to the broth at 2–512 μg/ml. Set control and blank group, and the surrounding wells were coated with phosphate buffered saline (PBS) to prevent dryness. Placed the plates in the incubator at 37°C for 16 h, and the absorbance at 600 nm was measured using a microplate reader (Thermo Fisher, United States).

### Growth assay

Growth curves were used to assess whether 2,3-DHKV influenced the growth of USA300. *S. aureus* USA300 was resuspended in TSB at a ratio of 1:100 and incubated overnight. Then, fresh TSA medium was added to adjust the *S. aureus* culture to an OD_600_ of 0.1, and then 64 μg/ml 2,3-DHKV or DMSO was added. The growth of *S. aureus* USA300 within 24 h was measured and the growth curve was drawn at a time interval of 1 h.

### Cell viability assay

The toxicity of 2,3-DHKV on A549 cells was assessed using the MTT assay (Van Meerlo et al., 2011). After routinely digested and counted, cells were plated at a density of 5 × 10^3^ per well in a 96 well plate. 2,3-DHKV was given at doses of 0–32 μg/ml for another day of incubation. After that, each well was added with 0.5% MTT solution for 4 h at 37°C. The supernatant was removed and the precipitate was dissolved in DMSO solution for 20 min before being quantified with a microplate reader at 490 nm.

### Hemolytic assay

The effects of 2,3-DHKV on the hemolytic activity of *S. aureus* USA300, Newman and SA1B3G were investigated, respectively. The hemolytic activity was determined as described in previous literature (Duan et al., 2018). Overnight cultures of the *S. aureus* USA300, Newman and SA1B3G were inoculated 1: 100 into TSB medium and grown at 37°C. Different concentrations of 2,3-DHKV (0–32 μg/ml) were added to cultures of *S. aureus* with an initial OD_600_ of 0.3, and incubation was continued until late logarithmic growth. The supernatant of 100 μl of the above cultures was mixed with 25 μl of defibrinated rabbit blood RBCs and PBS to 1 ml. Triton X-100 instead of *S. aureus* culture supernatant was set as a positive control. For 1 h, the mixture was incubated at 37°C.The supernatant was subsequently centrifuged to remove the supernatant and the absorbance value of OD_543_ nm was measured. The Triton X-100 treated group was served as the 100% hemolysis control, and the percent hemolysis was calculated by comparison with the control culture (Jin et al., 2018).

### Neutralization activity

The overnight culture of *S. aureus* USA300 was diluted 1:100 into fresh medium and continued to incubate until OD_600_ reached 2.5. The culture supernatant was collected and treated with different concentrations of 2,3-DHKV (0–64 μg/ml). In a 1.5 ml EP tube, 25 μl of RBCs, 100 μl of supernatant of the culture and 875 μl of PBS were added, mixed and incubated at 37°C for 1 h. The precipitation was subsequently centrifuged to remove. Then, the absorbance of the supernatant was measured at 543 nm. The assays were performed in triplicate.

### Heptamer formation

To investigate whether 2,3-DHKV affects the formation of Hla oligomerization, we performed a heptamer formation assay as previously described (Wang et al., 2016, 2020). Firstly, purified Hla proteins (20 μg) and different concentrations of 2,3-DHKV (0–128 μg/ml) were mixed with PBS and incubated at 22°C for 25 min. The sample was then incubated at 55°C for 10 min with 5 × protein loading buffer without β-mercaptoethanol (β-ME). The incubated solutions were subjected to 8% SDS-PAGE. Banding images were acquired after Coomassie blue staining.

### Cellular thermal shift assay

Cellular thermal shift assay was employed to examine the interaction between 2,3-DHHKV and Hla, as previously described (Ren et al., 2022). First, the expression of Hla protein in *E. coli* BL21 expressing hosts containing pET28a-hla was induced. The samples were equally split into two copies, one treated with 2,3-DHKV (64 μg/ml) and the other with DMSO at 37°C for 1 h in the dark. After centrifugation at 12,000 rpm × 20 min at 4°C, the supernatant was collected and separated into PCR tubes, setting the specific temperature (25°C, 45°C, 51.2°C, 56.2°C, 61.4°C, 65°C) of the Hla protein, and placed in a Mastercycler^®^ neus gradient (Eppendorf) for heating. After heating for 5 min, they were immediately cooled on ice for 3 min. Then samples were analyzed by SDS-PAGE, and bands were developed after destaining the stain.

### Real-time quantitative PCR

Real-time quantitative PCR was performed to further analyze the transcription levels of *hla*, *agrA*, Panton-Valentine leucocidin (*PVL*), phenol-soluble modulin alpha (*psmα*) and seven serine protease A (*sspA*) in the presence of various concentrations of 2,3-DHKV. A culture of *S. aureus* with an initial OD_600_ of 0.3 was added with 2,3-DHKV (0–16 μg/ml) or DMSO, and was shaken and incubated until the OD_600_ was 2.5. After centrifugation at 12,000 rpm for 1 min, the supernatant was discarded. The bacteria were subsequently collected and total RNA from the co-cultures was extracted with TRIzol solution (Tiangen, Beijing, China). Subsequently, 1 μg of purified RNA was used to generate cDNA using the HiScript^®^ II 1st Strand cDNA Synthesis Kit (#R211, Vazyme, Nanjing, China) and stored at-20°C. Next, qPCR was performed with the following cycle parameters: 95°C for 30 s, 40 cycles of 95°C for 5 s and 60°C for 30 s and 72°C for 30 s on an ABI 7900HT Real-time PCR system. The *16sRNA* was used as an internal reference gene. The 2^−ΔΔCT^ method was used to calculate the relative transcript levels of the gene *hla*, *agrA*, *PVL* and *psmα*. Table 1 lists the primers for these genes.

### Western blot

Different concentrations of 2,3-DHKV (0–32 μg/ml) were added to the suspension of *S. aureus* with an initial OD_600_ of 0.3 and incubated with shaking until late logarithmic growth. Subsequently, the supernatant was collected and immediately subjected to 12% SDS-PAGE, followed by the proteins was transferred to the PVDF membrane *via* a semi-dry transfer apparatus. The membranes were blocked for 2 h at room temperature with 5% bovine serum albumin (BSA), followed by three washes with PBST. Next, the protein membranes were immunoreacted with rabbit anti-Hla antibody (1:3000, Sigma-Aldrich) and HRP labeled goat anti-rabbit IgG (1:800, Sigma-Aldrich), respectively. After washing the membrane for 3 times, Super ECL Plus (S6009M, US EVERBRIGHT Suzhou, China) was added to visualize the bands using an enhanced chemiluminescence (ECL) detection device.

### Infection of A549 cells and lactate dehydrogenase assay

A549 human lung epithelial cells were seeded into 24 well plates at 1 × 10^5^ per well and then incubated at 37°C in a 5% CO_2_ incubator for 24 h. When cell have grown to 80% confluence, remove the medium. The protective effect of 2,3-DHKV on *S. aureus*-infected A549 cells was verified by two different treatments. Then fresh medium, *S. aureus* USA300 suspension (50 μl, OD_600_ = 0.5) and various concentrations of 2,3-DHKV (0–32 μg/ml) were added to each well. Alternatively, overnight cultures of *S. aureus* were filled with fresh medium at a ratio of 1:100 with various concentrations of 2,3-DHKV and incubation was continued with shaking until OD_600_ reached 0.5. Subsequently, 50 μl of the above bacterial cultures were filled into the culture plates containing A549 cells. Untreated cells were used as a blank control group. The plates were placed in an incubator and continued to incubate for 6 h at 37°C, and the supernatant was collected to measure the release content of LDH using a LDH Kit (Beyotime, Beijing, China) at 490 nm.

Next, A549 cells (treatment groups with simultaneous addition of *S. aureus* and 2,3-DHKV) were subjected to Calcein/PI cell viability assay. The pretreated staining solution was added to the cells and incubated for half an hour in the dark. Cell staining was observed and photographed under a fluorescence microscope (Olympus, Japan).

### MRSA-infected pneumonia murine model

C57BL/6 J mice (7 weeks, about 22 g) were selected for the establishment of a mouse pneumonia infection model induced by *S. aureus*. The mice were supplied by Liaoning Changsheng Biotech Co., Ltd. (Changchun, China). After 1 week of adaptive feeding, mice were conducted nasal inhalation of USA300 to establish the pneumonia model and randomly divided into three groups, the USA300 infection group, 2,3-DHKV treated group and uninfected control group.

For survival analysis, mice were intranasally administered a suspension of *S. aureus* with 2 × 10^8^ CFUs. The treatment group was subcutaneously injected with 40 mg/kg 2,3-DHKV 1 h after infection, and then administered again every 12 h. The infected group was given an equal volume of normal saline (0.05% DMSO) in the same way. The survival of mice was observed and recorded every 12 h to72 h, and the corresponding survival curve was then drawn.

With the purpose of investigating the therapeutic effect of 2,3-DHKV, we further analyzed the bacterial load of lung tissues and the pathological changes in lung tissue. For bacterial load analysis, mice were nasally administered with USA300 (1 × 10^8^ CFUs) to conduct infection. The method of grouping was same as the survival assay mentioned above. After 48 h of infection, the mice were euthanized. Lung tissues were collected, weighed, and homogenized, and then the tissue was smeared onto TSA agar plates to incubate in a 37°C incubator, followed by colony counting after colony growth. The left lung from each mouse was taken and the changes of lung were observed. Subsequently, the left lung tissues of mice in each group were aseptically removed and fixed in 10% formalin. After conventional hematoxylin and eosin (H&E) staining, the lung tissue sections photographed and recorded under light microscopy. In addition, Hla levels in the alveolar lavage fluid of each group of mice were measured by western blot. Alveolar lavage fluid was obtained by flushing each mouse with 1.5 ml of saline, and western blot was performed after quantification by the BCA assay.

### Ethical statement

The animal study was reviewed and approved by Institutional Animal Care and Use Committee (IACUC) of Jilin University.

### Statistical analysis

Each test was performed three times, and data were expressed as mean ± SD, with *p* < 0.05 regarded statistically significant. Data analysis was carried out using GraphPad Prism 8.0 (GraphPad Software Inc., San Diego, United States). Two sample comparisons used the student’s *t*-test (normal distribution) or the Mann–Whitney U-test (abnormal distribution). Multiple groups of samples were compared using one-way ANOVA or nonparametric test. The log-rank test was used to determine the mouse survival curves.

## Results

### 2,3-DHKV inhibits the hemolytic ability of *S. aureus* without affecting bacterial growth

**Figure 1:**
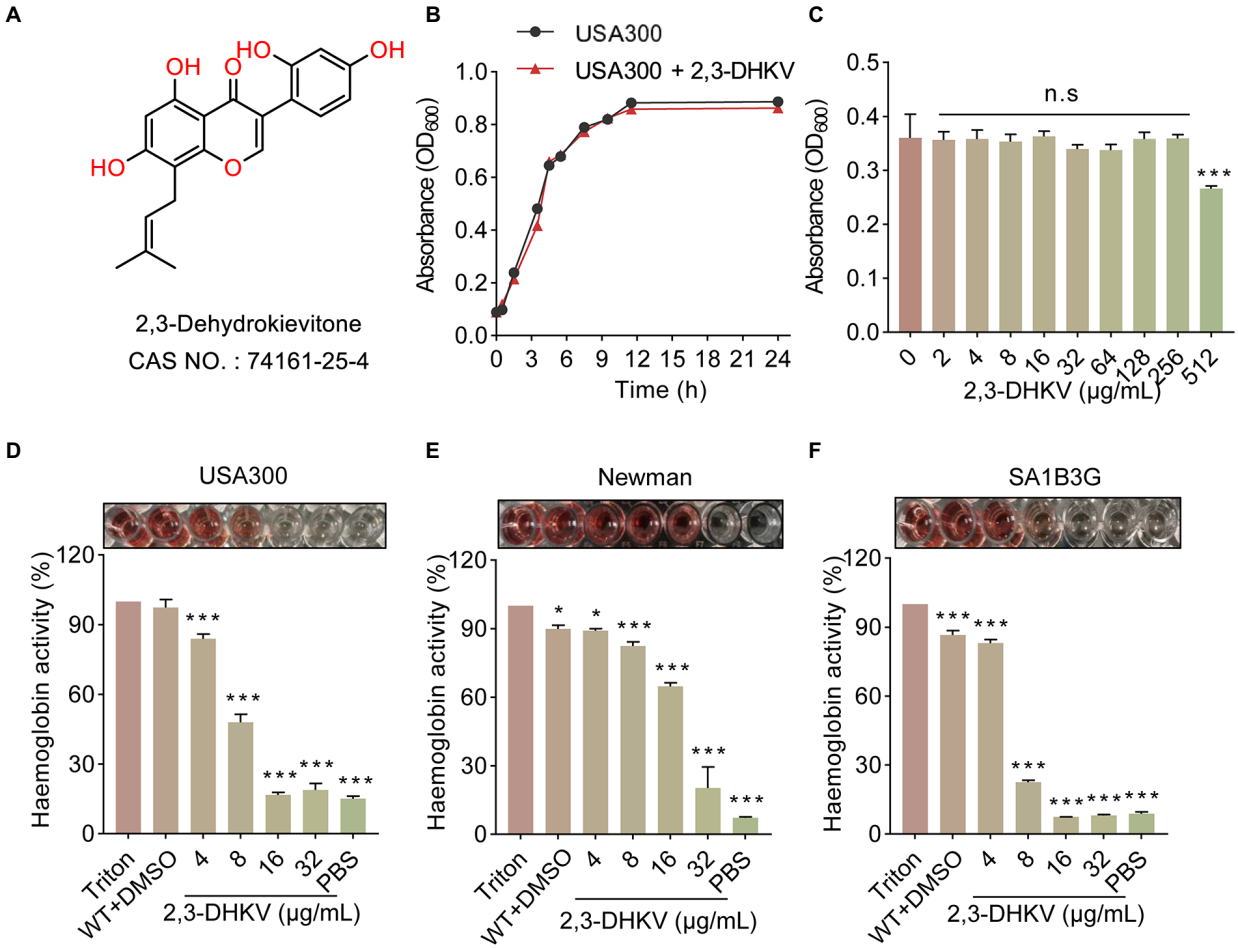
2,3-DHKV inhibits the hemolytic ability of *S. aureus* without affecting bacterial growth. **(A)** Chemical structure of 2,3-DHKV. **(B)** Growth curve of USA300 with or without 64 μg/ml 2,3-DHKV. **(C)** The MIC of 2,3-DHKV against USA300 by micro broth dilution assay. **(D–F)** The effects of 2,3-DHKV on the hemolytic activity of USA300, Newman, and SA1B3G. Triton X-100 was set as a negative control. Data are presented as mean ± SD (*n* = 3), ^*^p < 0.05, ^***^p < 0.001.

Antivirulence factors candidate drugs, unlike the bactericidal action of antibiotics, not only need to possess the ability to combat the virulence of bacteria but also do not interfere with *S. aureus* growth. 2,3-DHKV, a natural isoflavonoid, was screened to meet these two conditions. Its chemical structure is shown (Figure 1A). The growth curve showed that the USA300 treated with 64 μg/ml 2, 3-DHkV had the same growth trend as the native state growth of USA300 with no difference (Figure 1B). Meanwhile, the MIC of 2,3-DHKV against USA300 was 512 μg/ml as showed by micro broth dilution assay, indicating that 2,3-DHKV had no inhibitory activity against MRSA USA300 (Figure 1C).

Importantly, we observed that the hemolytic activity of *S. aureus* USA300, Newman and SA1B3G were dramatically decreased in a manner of dose-dependent under 2, 3-DHKV treatment (*p* < 0.05; Figures 1D–F). For *S. aureus* USA300, at 16 and 32 μg/ml 2,3-DHKV treatment caused a significant decrease in lysed RBCs, and the solution color was similar to that of the PBS-treated group, indicating the hemolytic activity was almost completely inhibited (Figure 1D). Similar results were observed in clinical isolates SA1B3G. Given the above facts, these results indicated that 2,3-DHKV can significantly inhibit the hemolytic activity of *S. aureus* without affecting bacterial growth.

### 2,3-DHKV does not affects the heptamer formation in *S. aureus*

**Figure 2:**
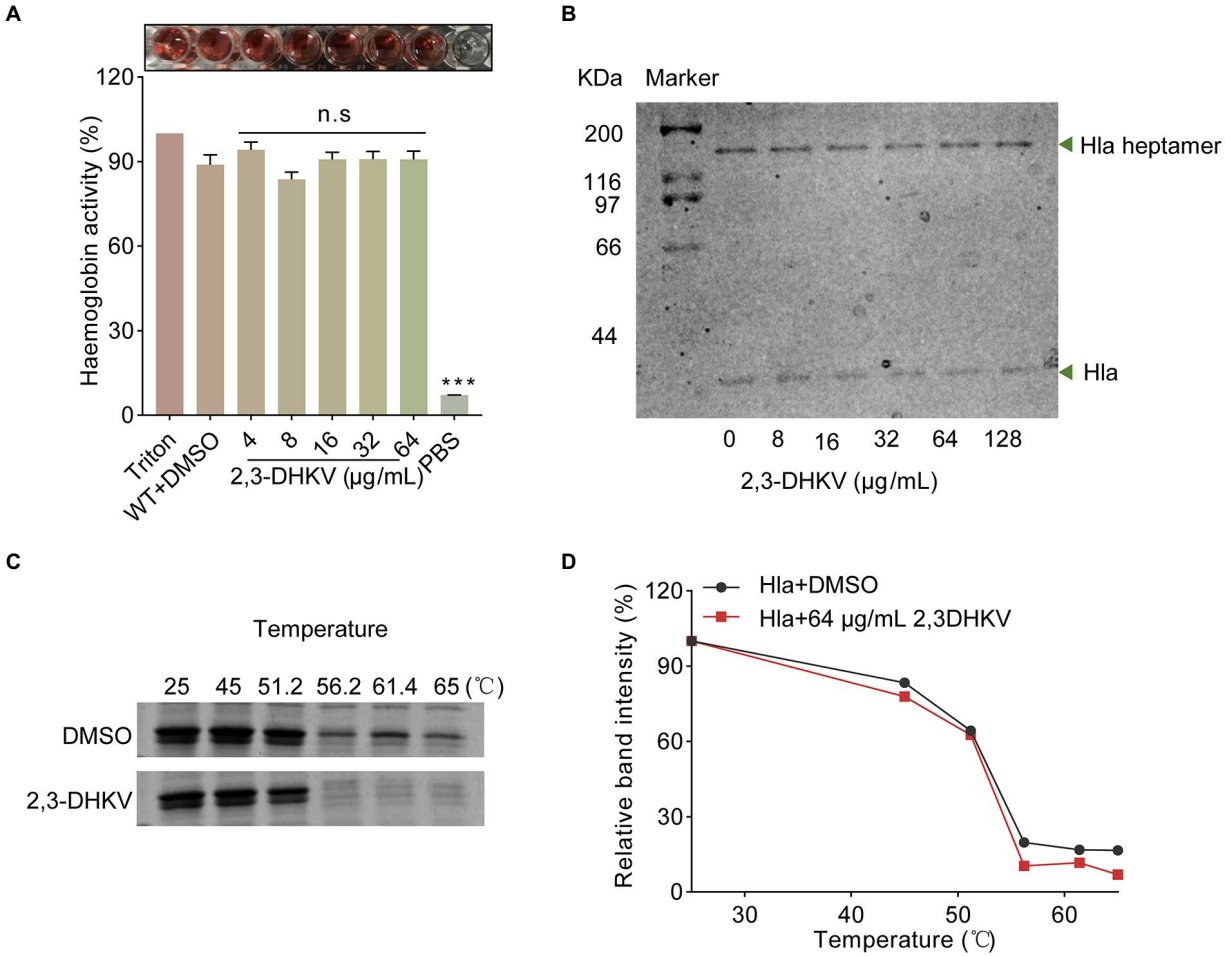
2,3-DHKV does not affect the heptamer formation of Hla in *S. aureus.* **(A)** The effect of 2,3-DHKV on the neutralization activity of Hla. **(B)** The heptamer formation assay of Hla treated or non-treated with 2,3-DHKV. **(C,D)** CETSA analysis was performed to analyze the direct interaction between 2,3-DHKV (64 μg/ml) and Hla.

To investigate the mechanism of Hla inhibition by 2,3-DHKV, neutralization activity of *S. aureus* was performed. The hemolytic assay revealed that 2,3-DHKV had no neutralizing activity against hla in the supernatant of *S. aureus* (Figure 2A). In addition, CETSA assays detected the direct drug-target interactions by quantifying the changes in the thermal stability of proteins upon ligand binding in intact cells (Asawa et al., 2020). To this end, we performed CETSA for binding effects of the natural products 2,3-DHKV and Hla. We found that in the 2,3-DHKV treatment group, the amount of Hla did not change significantly with increasing temperature compared to the DMSO group, indicating that 2,3-DHKV did not affect the thermal stability of Hla (Figures 2C,D). Furthermore, with the increase of 2,3-DHKV, heptamer formation in the samples did not change significantly, suggesting that 2,3-DHKV did not inhibit hemolytic activity by affecting Hla heptamer formation (Figure 2B). Based on the above analysis, these results suggested that the inhibitory effect of 2,3-DHKV on *S. aureus* hemolysis is not achieved by direct binding to Hla.

### 2,3-DHKV downregulates the transcription of *Hla* and Agr system regulatory-related genes

**Figure 3:**
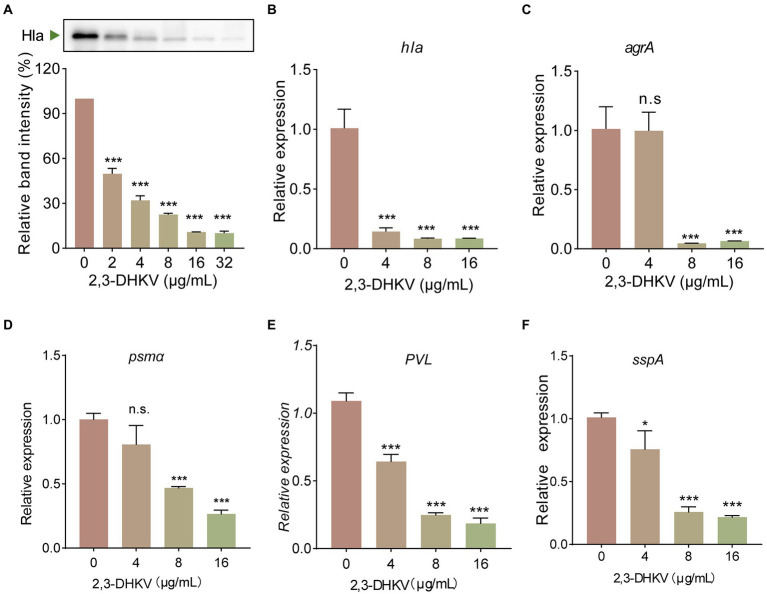
2,3-DHKV significantly decreases the expression of Hla and Agr system regulatory-related genes. **(A)** Expression levels of Hla in USA300 after treatment with different concentrations of 2,3-DHKV (0–32 μg/ml) were determined by western blot. Transcription level of **(B)** *hla*, **(C)** *agrA*, **(D)** *psmα*, **(E)** *PVL* and **(F)** *sspA* were determined in USA300 treated or untreated with 2,3-DHKV, and 16sRNA was used as an internal reference gene. Data are presented as mean ± SD (*n* = 3), n.s., no significant difference; ^*^p < 0.05; ^***^p < 0.001.

2,3-DHKV showed significant anti-hemolytic potency without direct binding to Hla. We next tested whether 2,3-DHKV inhibited hemolysis activity by reducing Hla production *via* RT-qPCR and western blot. Figure 3A showed that 2,3-DHKV significantly reduced the level of Hla protein expression in a concentration-dependent manner (*p* < 0.001), as judged by the 49.8% at 2 μg/ml decreased to 10.1% at 32 μg/ml. Furthermore, at the mRNA level, 2,3-DHKV similarly significantly reduced *hla* transcription at 16 μg/ml (*p* < 0.001), which was eleven-fold lower than WT group (Figure 3B). Subsequently, we further examined the effects of 2,3-DHKV on the transcription level of the upstream regulatory gene *agrA*, and the results also suggested that 2,3-DHKV could significantly inhibit *agrA* transcription (*p* < 0.001; Figure 3C). *Psmα* and PVL can destroy a variety of host cells and are essential to *S. aureus* infection (Shallcross et al., 2013). We investigated the production of *psmα* and PVL in the presence of different concentrations of 2,3-DHKV. The transcription levels of *psmα* and *PVL* were decreased to different degrees in 2,3-DHKV-treated *S. aureus* and exhibited a dose dependence (Figures 3D,E). In addition, sspA, an extracellular protein hydrolase of *S. aureus*, was also observed to significantly decrease transcript levels after treatment with 2,3-DHKV (Figure 3F). These results suggested that 2,3-DHKV may affect its downstream regulatory genes by affecting the Agr system and thus its downstream regulatory genes.

### 2,3-DHKV protects *S. aureus*-mediated A549 cell injury

**Figure 4:**
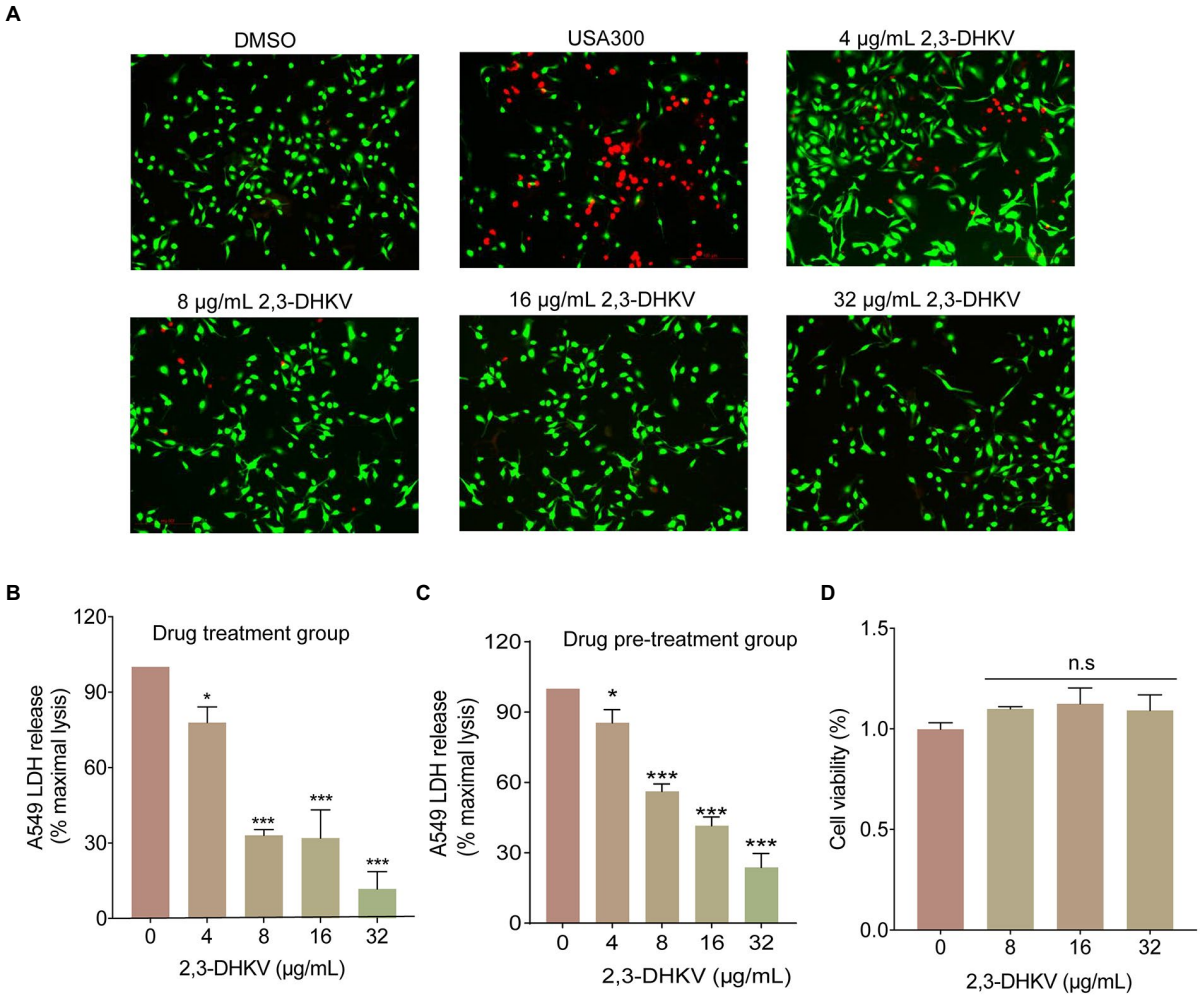
2,3-DHKV protects A549 cells from infection-induced damage. **(A)** A549 cells were infected with USA300 and treated with 2, 3-DHKV for live (green)/dead (red) cell staining assay. The LDH release was applied to evaluate the cytotoxicity of various concentrations of 2, 3-DHKV to supernatant of *S. aureus* USA300 infected A549 cells. **(B)** 2, 3-DHKV with *S. aureus* simultaneous addition group (Drug treatment group) **(C)** 2, 3-DHKV with *S. aureus* mixed culture group (Drug pre-treatment group). **(D)** MTT assay was used to assess the effect of 2,3-DHKV on the viability of A549 cells. ^*^p < 0.05, ^***^p < 0.001.

A549 cells infected with *S. aureus* USA300 were treated with 2, 3-DHKV to briefly mimic the therapeutic impact of 2,3-DHKV after MRSA invasion. As shown in Figure 4A, cells in the DMSO group exhibited almost exclusively green fluorescence (live cells). After infection with USA300, a large amount of red fluorescence was observed, and the cell morphology was suboptimal with the increase in dead cells. After treatment with different concentrations of 2,3-DHKV (0–32 μg/ml), the red fluorescence of A549 cells was decreased, demonstrating that 2,3-DHKV reduced cell damage resulting from USA300 infection (Figure 4A). Furthermore, LDH is released extracellularly after cell injury, and its content is proportional to the number of cell lyses (Cardoso et al., 2018), which is one of the indicators for evaluating cell survival. We performed different drug treatments to assess the protective effect of 2,3-DHKV on A549 cells infected with *S. aureus*, including 2,3-DHKV pre-treatment with *S. aureus* and 2,3-DHKV added simultaneously with *S. aureus* to plates containing A549 cells. By detecting LDH content in each group, compared with USA300 infection group 2, 3-DHKV significantly reduced LDH release (*p* < 0.05), implying that it reduced MRSA attack on A549 cells (Figures 4B,C).

In addition, the MTT assay was observed that 2,3-DHKV did not affect the viability of A549 cells at the concentration of inhibiting hemolytic activity, indicating that 2,3-DHKV was relatively safe (Figure 4D). The above findings supported our view that 2,3-DHKV protected A549 cells from *S. aureus*-induced injury to a certain extent.

### 2,3-DHKV protects mice from *S. aureus* pneumonia infection

**Figure 5:**
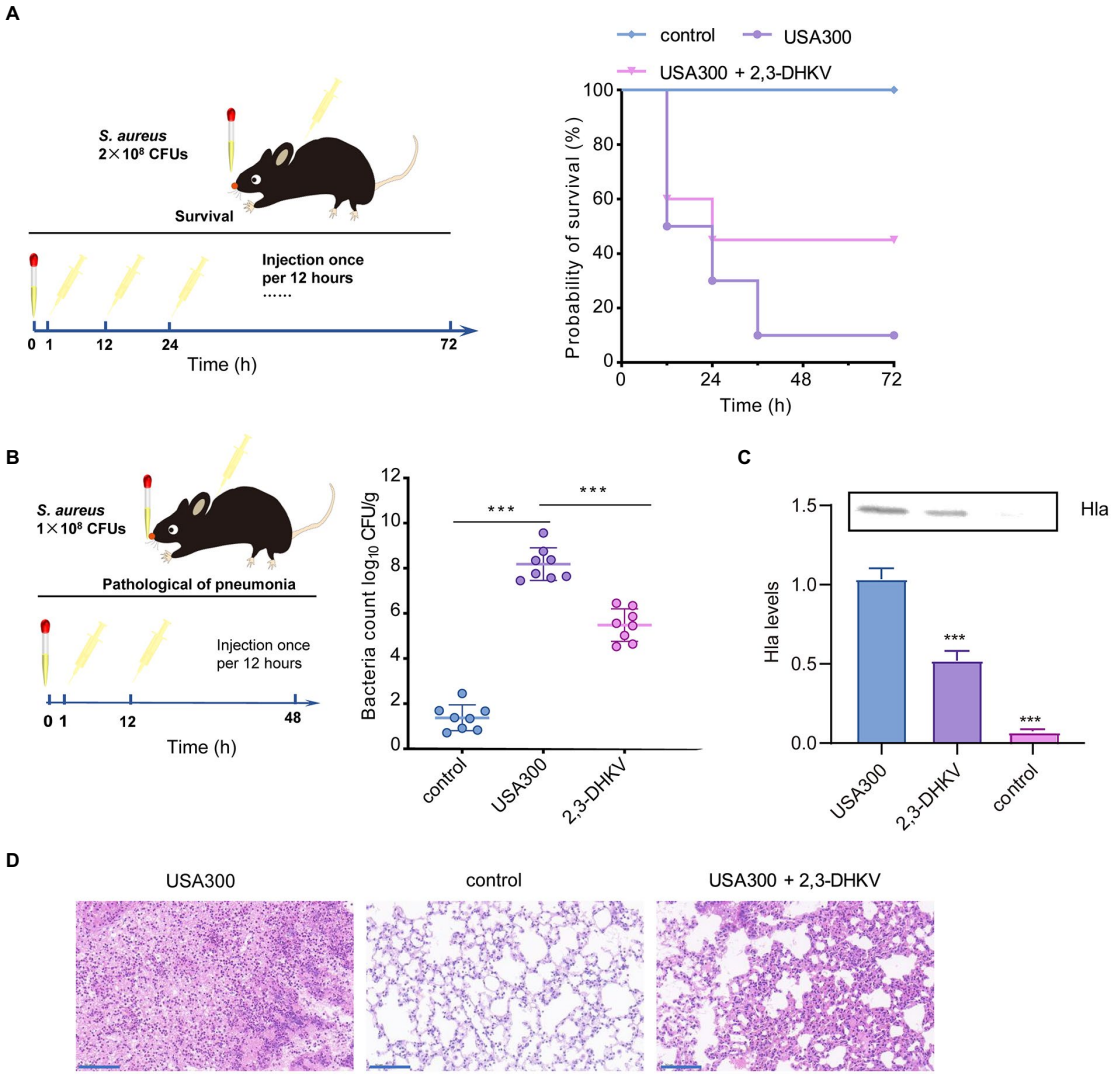
2,3-DHKV protects against *S. aureus* pneumonia infection in mice. **(A)** The protective effect of 2,3-DHKV on infected mice was evaluated based on the survival rate that was recorded at 12 h intervals for 72 h (*n* = 10), and the uninfected group acted as a positive control. **(B)** The bacterial load of murine lungs treated with or without 40 mg/kg 2,3-DHKV, and the uninfected group served as a positive control (*n* = 8). **(C)** Hla level in alveolar lavage fluid of mice treated or untreated with 2,3-DHKV after infection with *S. aureus* USA300. **(D)** Histopathological analysis (H&E staining) of *S. aureus*-infected mouse lungs with or without 2,3-DHKV treatment. Scale bar, 100 μm. The data is provided as mean ± SD, ^***^p < 0.001.

We further demonstrated the protective role of 2,3-DHKV *in vivo* by establishing a lethal MRSA infection pneumonia model. A mouse pneumonia infection model was established by nasal drip administration of *S. aureus* suspension. Subsequently, 2,3-DHKV was administered subcutaneously at 1 h of infection and every 12 h interval for treatment (Figure 5A). The survival rate of the mice within 72 h after infection was 10%, indicating that the pneumonia infection model was successfully established. When treated with 2,3-DHKV, we observed an increase in the survival rate of mice from 10% to 50% (Figure 5B), suggesting that treatment of pneumonic infection in mice with 2,3-DHKV could significantly improve survival rates, particularly in the early stages of infection.

The bacterial load of the lung tissue of each group of mice was further evaluated. As exhibited in Figure 5B, the USA300 group was implanted with 8.18 ± 0.72 log_10_ CFUs/g bacteria, whereas the 2,3-DHKV group significantly reduced it to 5.49 ± 0.72 log_10_ CFUs/g (*p* < 0.001). Furthermore, we further measured the changes in Hla levels in the alveolar lavage fluid of mice in each group. As expected, Hla was not detected in the alveolar lavage fluid of the control group. Compared to the infected group, mice treated with 2,3-DHKV significantly reduced the level of Hla in the alveolar lavage fluid (Figure 5C).

In addition, we observed the pathological changes in each group of mice. There was a large amount of inflammatory cell infiltration in the lungs of mice in the infection group, and treatment with 2, 3-DHKV significantly reduced inflammation by reducing the accumulation of inflammatory cells in the alveolar space (Figure 5D).

Overall, 2,3-DHKV exhibited a strong protective impact against *S. aureus*-induced fatal pneumonia in mice, enhancing the survival rate of mice, lowering the number of colonies in the lungs, and alleviating lung tissue damage.

## Discussion

MRSA, a notorious hospital dweller, has placed a heavy burden on patients in long-term intensive care. In clinical practice, clinicians are often confronted with various diseases caused by MRSA infection. Currently, the first-line treatment of MRSA is vancomycin, although the treatment is quite restrictive, especially with the emergence of vancomycin-resistant *Staphylococcus aureus* (VRSA) at the end of the 20th century, undoubtedly causing a great shake and crisis in the antibiotic treatment of MRSA (Gao et al., 2021). Thus, changing treatment strategies may find a way out of this dilemma. In addition to traditional antibiotics, the development of drug replacement therapies that reduce the pathogenic capacity of MRSA, such as quorum sensing inhibitors, therapeutic antibodies targeting virulence factors and bacteriophages, is expected to make vital contributions to the treatment of *S. aureus* disease (Vuong et al., 2016).

Hla, the most representative virulence factor that largely contributes to *S. aureus* pathogenicity, is an extracellular toxin secreted by 95% of *S. aureus* (Grumann et al., 2014). The toxicity of Hla is very high, with an injection of 1 μg into rabbit blood causing death immediately (Dinges et al., 2000). Hla monomers associate with the cognate receptor zinc-dependent metalloproteinase A disintegrin and metalloproteinase 10 (ADAM10) to exert activity and further oligomerize into heptamers with pore-forming on the host cell membrane, which caused cell swelling and rupturing (Vuong et al., 2016). Thus, inhibition of hemolysin expression would lead to a decrease in heptamer formation and thus reduce hemolytic activity. Monoclonal antibodies (mAbs) targeting Hla have been developed to combat *S. aureus* infection, such as MEDI4893, which interferes with hemolytic function by inhibiting heptamer formation and interfering with ADAM10 interactions (Ragle and Bubeck Wardenburg, 2009; Foletti et al., 2013; Oganesyan et al., 2014). Moreover, Hla could alter the platelet morphology and hence may influence the thrombotic events associated with *S. aureus* sepsis (Divyakolu et al., 2019). This study found that flavonoid 2,3-DHKV could strongly reduce *S. aureus*’ hemolytic activity in this study. Some natural compounds, such as myricetin and baicalin, could directly bind Hla and interfere with the self-assembly process (Qiu et al., 2012; Silva et al., 2017). It has also been reported that some natural products could inhibit the expression of Hla and thus inhibit hemolytic activity, such as farrerol (Qiu et al., 2011), resveratrol (Tang et al., 2019), etc. In our study, 2,3-DHKV exerted the latter effect, neither affecting the formation of aggregates nor the direct interaction with Hla, but achieving the anti-hemolysis effect by reducing the expression of hemolysin.

In *S. aureus*, the accessory gene regulator (Agr) quorum-sensing system can regulate Hla production (Singh and Ray, 2014). For example, dracorhodin inhibits the Agr quorum-sensing system and Hla production in *S. aureus* (Liu et al., 2017). To this end, we used RT-qPCR to confirm that 2,3-DHKV significantly downregulated *agrA* transcription levels, suggesting that reduced Hla expression may be related to Agr population-sensing regulation. Among them, *RNAIII* is an important regulatory factor in the agr system. *RNAIII* regulates the expression of multiple virulence genes, such as hemolysins, enterotoxins, and proteases (Kane et al., 2018). It also regulates a number of cell surface-associated proteins including fibronectin-binding proteins and protein A. In addition to *RNAIII*, the AgrA response regulator can also regulate phenol-soluble modulins (PSM) alpha and beta by directly binding the promoter regions (Singh and Ray, 2014). Our experiments also confirmed that 2,3-DHKV may affect the Agr system by influencing its downstream regulatory genes. However, whether 2,3-DHKV works by directly affecting Agr or by impacting other systems still needs further elucidation. It has also been reported that Agr is activated upon phagocytosis of USA300 MRSA leading to the upregulated expression of the Hla within the phagosome and contributing to neutrophil destruction in as little as 2 h, which is conducive to *S. aureus* immune escape (Nizet, 2010).

Subsequently, 2,3-DHKV was added to the co-culture system of *S. aureus* and alveolar epithelial cells A549, and the content of LDH was detected by fluorescence microscope. The results showed that 2,3-DHKV significantly protected against A549 cell damage mediated by *S. aureus*, suggesting a potential therapeutic effect of 2,3-DHKV on *S. aureus* pneumonia. In addition, Bubeck et al (Bubeck et al., 2007) demonstrated a central role for Hla in *S. aureus* pneumonia. In a mouse model of *S. aureus* pneumonia, the deletion of Hla deficient strains was significantly lower than the pathogenicity and lethality of normal strains. McElroy et al (McElroy et al., 1999) established a model of *S. aureus* pneumonia by intranasal instillation in rabbits, proving that Hla could damage the air-blood barrier in the lungs, causing inflammatory cells to enter the alveolar space. In fact, we also observed consistent effects that 2,3-DHKV protected mice from lethal MRSA pneumonia infection, improving survival, reducing bacterial colonization and attenuating lung tissue damage.

In conclusion, the natural product 2, 3-DHKV exhibited antibacterial efficacy against MRSA by inhibiting hemolytic activity *in vitro* and *in vivo* without significant cytotoxicity, providing feasibility for further study of candidate agents for MRSA infection.

## Data availability statement

The original contributions presented in the study are included in the article/supplementary material, further inquiries can be directed to the corresponding author.

## Ethics statement

The animal study was reviewed and approved by the animal study was reviewed and approved by Institutional Animal Care and Use Committee (IACUC) of Jilin University.

## Author contributions

The material preparation and the data collection and analyses were performed by HY, JL, and SG. The first draft of the manuscript was written by HY and LW. DW revised the manuscript. HX and DW conceived of the study and designed the experiments. All authors contributed to the article and approved the submitted version.

## Funding

This work was supported by the Science Foundation of Jilin Province, China (No. 20220101286JC), Science Foundation of Jilin Province, China (No. 20180101276JC), and the National Key Research and Development Program of China (No. 2018YFD0500300).

## Conflict of interest

The authors declare that the research was conducted in the absence of any commercial or financial relationships that could be construed as a potential conflict of interest.

## Publisher’s note

All claims expressed in this article are solely those of the authors and do not necessarily represent those of their affiliated organizations, or those of the publisher, the editors and the reviewers. Any product that may be evaluated in this article, or claim that may be made by its manufacturer, is not guaranteed or endorsed by the publisher.
